# Airway hyperresponsiveness is associated with airway remodeling but not inflammation in aging *Cav1*^*-/-*^ mice

**DOI:** 10.1186/1465-9921-14-110

**Published:** 2013-10-21

**Authors:** Kelsa E Gabehart, Simon G Royce, Diego J Maselli, Shelley K Miyasato, Elaine C Davis, Mimi LK Tang, Claude Jourdan Le Saux

**Affiliations:** 1 Department of Cell and Molecular Biology, John A. Burns School of Medicine, University of Hawaii, Honolulu, HI, USA; 2 Department of Allergy and Immune Disorders, Murdoch Children’s Research Institute, Parkville, Melbourne, Victoria, Australia; 3 Department of Medicine, Division of Cardiology and Pulmonary and Critical Care, The University of Texas Health Science Center, San Antonio, TX, USA; 4 Department of Anatomy and Cell Biology, McGill University, Montreal, Quebec, Canada; 5 Department of Allergy and Immunology, The Royal Children’s Hospital, Melbourne, Victoria, Australia; 6 Department of Pediatrics, The University of Melbourne, Parkville, Melbourne, Victoria, Australia

## Abstract

**Background:**

Airway inflammation and airway remodeling are the key contributors to airway hyperresponsiveness (AHR), a characteristic feature of asthma. Both processes are regulated by Transforming Growth Factor (TGF)-β. Caveolin 1 (Cav1) is a membrane bound protein that binds to a variety of receptor and signaling proteins, including the TGF-β receptors. We hypothesized that caveolin-1 deficiency promotes structural alterations of the airways that develop with age will predispose to an increased response to allergen challenge.

**Methods:**

AHR was measured in Cav1-deficient and wild-type (WT) mice 1 to 12 months of age to examine the role of Cav1 in AHR and the relative contribution of inflammation and airway remodeling. AHR was then measured in *Cav1*^*-/-*^ and WT mice after an ovalbumin-allergen challenge performed at either 2 months of age, when remodeling in *Cav1*^*-/-*^ and WT mice was equivalent, and at 6 months of age, when the *Cav1*^*-/-*^ mice had established airway remodeling.

**Results:**

*Cav1*^*-/-*^ mice developed increased thickness of the subepithelial layer and a correspondingly increased AHR as they aged. In addition, allergen-challenged *Cav1*^*-/-*^ mice had an increase in AHR greater than WT mice that was largely independent of inflammation. *Cav1*^*-/-*^ mice challenged at 6 months of age have decreased AHR compared to those challenged at 2 months with correspondingly decreased BAL IL-4 and IL-5 levels, inflammatory cell counts and percentage of eosinophils. In addition, in response to OVA challenge, the number of goblet cells and α-SMA positive cells in the airways were reduced with age in response to OVA challenge in contrast to an increased collagen deposition further enhanced in absence of Cav1.

**Conclusion:**

A lack of Cav1 contributed to the thickness of the subepithelial layer in mice as they aged resulting in an increase in AHR independent of inflammation, demonstrating the important contribution of airway structural changes to AHR. In addition, age in the *Cav1*^*-/-*^ mice is a contributing factor to airway remodeling in the response to allergen challenge.

## Introduction

Asthma is a major public health problem, affecting patients of all ages from infants to older adults. Morbidity and mortality are greater in older patients (> 65 yrs) despite a similar prevalence as that in younger asthmatics, and yet there is limited evidence on how age can influence the pathogenesis of asthma [1,2]. Older patients have a larger than predicted reduction in pulmonary function parameters even though physician-assessed severity, duration of diagnosed asthma, and smoking status were not different compared to younger adults. A significant increase in the comorbid diagnosis of chronic obstructive pulmonary disease is associated with asthma in older patients suggesting that long-standing asthma may lead to irreversible airflow obstruction [3]. Asthma is characterized by airway hyperresponsiveness (AHR), an exaggerated narrowing of the airway in response to stimuli. AHR can reflect asthma severity, and has been associated with a variety of contributing factors [4-7]. AHR has two distinct components. First, a transient component occurring after an allergen exposure that is linked to acute inflammation, second, a more persistent component, associated with the chronicity of the disease causing structural changes in the airways known as airway remodeling. Several features of airway remodeling can contribute to AHR, including: increased sensitivity, hypertrophy and hyperplasia of smooth muscle cells (SMCs), as well as increased subepithelial fibrosis characterized by increased collagen deposition [8]. The development of structural changes persists during and after acute inflammation in allergic asthma subjects and in allergen challenge animal models with low inflammation [9-11]. Some asthma patients develop AHR in association with airway remodeling [12]. Furthermore, severe asthmatic patients under anti-inflammatory treatments will present loss in lung function despite inflammation being controlled [13]. These facts illustrate that underlying non-inflammatory mechanisms regulate airway structure and function, which include epithelial thickening and subepithelial collagen deposition.

Caveolin 1 (Cav1) is the main structural and functional protein of caveolae. These are 10 to 100 nm wide invaginations of the plasma membrane found in many cell types. Cav1 acts as a scaffolding protein, as well as a regulatory protein in many signaling cascade protein complexes. Cav1 inhibits the activity of these signaling proteins by binding and releasing them in a controlled fashion [14]. Cav1 has been shown to be involved in the regulation of both inflammation and fibrosis [15-19]. One of the important pathways that Cav1 regulates is the Transforming Growth Factor (TGF)-β pathway. Cav1 binds to and inhibits the TGF-β type II receptor thus preventing downstream signaling including the phosphorylation of Smad2/3. Others and our group have demonstrated that *Cav1*^*-/-*^ mice have enhanced Smad2 phosphorylation and altered ECM deposition notably in the lungs [18,19]. We have shown that aged *Cav1*^*-/-*^ mice have decreased lung function (i.e. increased elastance and decreased compliance) mainly due to an increase in ECM deposition of collagen and elastin [19]. The regulation of Cav1 has also been shown to be important in allergic airway disease [20,21]. We have previously shown that TGF-β signaling is enhanced in ovalbumin (OVA) challenged *Cav1*^*-/-*^ mice leading to enhanced airway remodeling [21].

TGF-β1 is a pleiotrophic growth factor that participates in resolution of inflammation, as well as promotion of airway remodeling, especially promoting extracellular matrix (ECM) deposition. Anti-TGF-β1 antibody treatment prevents the progression of airway remodeling following allergen challenge in mice [22]. More surprisingly, enhanced AHR has been reported in anti-TGF-β antibody treated OVA challenged mice associated with reduced Smad2 phosphorylation, marker of canonical TGF-β signaling pathway activation [23]. The regulation of TGF-β signaling activity has been the focus of intensive studies as a potential therapeutic target but it has not yet been fully characterized.

This study was designed to evaluate the relationship between inflammation, structural changes, and lung function in aged and OVA-challenged mice. We hypothesized that structural alterations of the airways that develop with age in Cav1 deficient mice will predispose them to an increased response to allergen challenge. The effect of Cav1-deficiency on airway structure was investigated, and AHR was associated to change in airway structures.

## Material and methods

### Animals and OVA-allergen challenge

Wild type C57Bl/6 J (WT) and Cav^tm1Mls/J^ (*Cav1*^*-/-*^*)* mice were purchased from The Jackson Laboratory (Bar Harbor, Maine) to establish our colonies and obtained the appropriate number of animals needed for our experiment. Animal experiments were conducted using a protocol approved by the Institutional Animal Care and Use Committee of the University of Hawaii.

Two- or 6-month-old *Cav1*^*-/-*^ and WT female mice were sensitized by 2 intraperitoneal (IP) injections of OVA absorbed on alum 12 days apart, followed 2 weeks later by intranasal challenge with OVA three times over a week. Measurement of acetylcholine induced AHR and sacrifice were performed 24 h after the last challenge as described previously [19]. A group of 2-month old mice was treated with the Cav1 scaffolding domain or scrambled peptide for 4 weeks as previously described [24].

### Measurement of airway hyperresponsiveness (AHR)

AHR was measured using a Flexivent instrument (Scireq Inc., Montreal, Quebec, Canada) as previously described [20]. Briefly, mice were anesthetized with ketamine (100 mg/kg IP) and xylazine (10 mg/kg IP). The trachea was then surgically exposed and cannulated to allow ventilation via the Flexivent at 150 breaths/min and a tidal volume of 7.5 ml/kg. Pancuronium bromide was administered IP to induce paralysis**.** Mice were then given increasing doses of acetylcholine (0.03, 0.1, 0.3, 1.0, 3.2 μg/g) in volume of 2 μl/g of body weight by tail vein administration at three-minute intervals and total lung resistance was measured to generate a dose-response curve. Log PC200 is the log of the dose required to increase resistance 200% over saline baseline. The lower the LogPC200 value the greater the reactivity [25].

### Sample collection

After completion of the AHR measurement, mice were disconnected from the animal ventilator and exsanguinated by cardiac puncture. Immediately after, bronchoalveolar lavage (BAL) was performed. Right side lobes were used for histology and electron microscopy (EM), and sections were cut longitudinally. EM preparation was carried out as previously described [19]. The left side lobes was reserved for RNA and protein extraction.

### Morphometric assessment

Photomicrograph images were captured using a Spot Cooled Color Digital camera (Q Imaging, Burnaby, BC, Canada). Briefly, a minimum of 5 bronchi, measuring 150–350 μm in luminal diameter with a continuous smooth muscle layer, were analyzed per mouse for the parameters described below, using Image Pro-Discovery software (Media Cybernetics, Silver Spring, MD), calibrated with a reference micrometer slide [10,26,27]. The thickness of the bronchial epithelial layer and subepithelial deposition of collagen were measured in sections stained with Masson’s trichrome.

### Morphometric α-SMA detection

Formalin-fixed paraffin-embedded sections were stained for α-smooth muscle actin (α-SMA) using immunohistochemistry and analyzed morphometrically. Primary antibody, mouse monoclonal anti α-SMA (Dako, Glostrup, Denmark), and a FITC labeled secondary antibody was used to detect α-SMA. The thickness of labeled α-SMA per length of basement membrane measured in a minimum of 5 bronchi, measuring 150–350 μm in luminal diameter, were analyzed per mouse for the parameters described below, using Image Pro-Discovery software (Media Cybernetics, Silver Spring, MD). Airways were selected and analyzed morphometrically for airway smooth muscle thickness using the method described above.

### Determination of airway inflammation

BAL was collected by injecting and withdrawing one ml cold phosphate-buffered saline (PBS). Recovered BAL (70–80%) was centrifuged at 300 *g* for 10 min. The fluid was collected for cytokine analyses. Cells were resuspended in 0.5 ml of PBS and an aliquot was removed to determine cell concentration using a hemocytometer. The remaining cells were adhered to glass slides using cytocentrifugation. Slides were stained using Wright-Giemsa stain and differential cell counts (minimum of 300 total cells) were obtained using a Zeiss light microscope. Cytokine levels were measured in BAL fluid using a Mouse Th1/Th2 Cytokine Kit Cytometric Bead Array™ (BD Biosciences Pharmingen) according to the manufacturer’s instructions.

### Airway remodeling assessment

1) Detection of goblet cells: Paraffin-embedded sections were deparafinized and stained using the standard procedure of periodic acid Schiff staining as described by the manufacturer (NovaUltra PAS Stain Kit, IHC WORLD, Woodstock, MD). We selected airways of approximately 100-200 μm in diameter and counted goblet cells in each airway. Five airways per animals were counted and a total of 3 animals per group were randomly selected.

2) *Detection of collagen deposition:* Paraffin-embedded lung sections were stained with *picrosirius red (PSR) stain*. Briefly, sections were deparafinized and hydrated to distilled water, then placed in phosphomolybdic acid 0.2% (cat. No 26356-01 Electron Microscopy Science, Hatfield, PA 19440) for 5 minutes, and placed directly in sirius red 0.2% in saturated picric acid (cat. No 26357-02; Electron Microscopy Science) for 90 minutes. Slides were washed in 0.01 N hydrochloric acid for 2 minutes and then placed in 70% ethanol for 45 seconds. Sections were dehydrated, cleared and mounted. PSR stained slides were quantified to determine the percent of collagen in the pulmonary tissue with Image Pro (Meyer Instruments Houston, TX 77084), a computer based analytical software. For each group, the quantification was performed on the entire upper right lobe of 5 mice.

3) *Detection of activation of the canonical TGF-β signaling pathway.* For analyses of levels of phospho-Smad2 (pSmad2) and total Smad2, whole lung homogenates were prepared in RIPA buffer in the presence of HALT^TM^ protease and phosphatase inhibitor cocktail (#78430 and # 78420, Thermo Fisher Scientific, Rockford, IL). All antibodies were purchased from Cell Signaling (Danvers, MA), except for β-actin (Cytoskeleton Inc; Denver, CO). Western blot analysis was carried out using the standard protocol as described previously [28]. Densitometric quantification was carried out by Image J software to represent the semi-quantitative protein levels. Relative levels of activated proteins were obtained as the ratio of phosphorylated protein: total protein levels. For detection of pSmad2 expression in allergen challenged lung tissue sections, non-inflated lung tissues were fixed in a solution of phosphate-buffered saline (PBS) containing 4% paraformaldehyde. Lungs were embedded in paraffin and sectioned. Following routine techniques of deparaffinization, antigen retrieval and quenching of peroxidase activity, the slides were incubated with primary pSmad2/3 antibody (cat# 9510S, Cell signaling, Danvers, MA) at a 1:500 dilution for 12 h, then biotinylated secondary antibody from VectaStain Elite ABC Mouse kit (PK-6102, Vector Laboratories Burlingame, CA 94010). We selected airways of approximately 100-200 μm in diameter and counted pSmad2/3 positive cells in each airway. Five airways per animals were counted and a total of 3 animals per group were randomly selected.

## Results

### ***Cav1***-deficient mice developed age-dependent airway hyperresponsiveness

**Figure 1:**
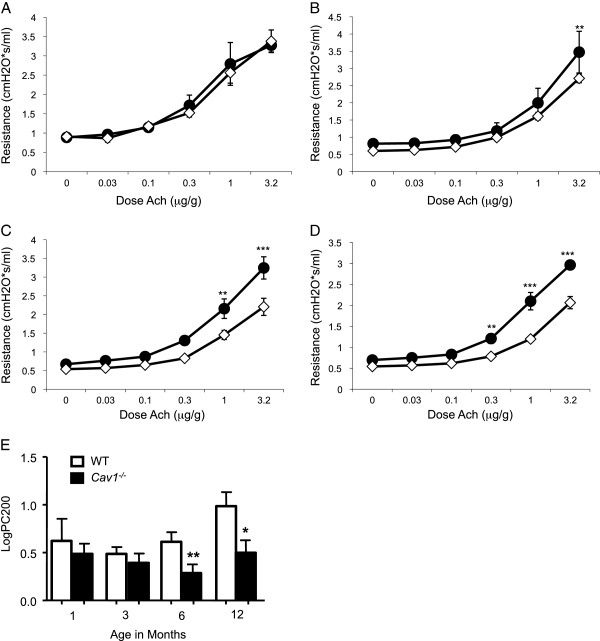
**Enhanced AHR in aging *Cav1***^**-/- **^**mice.** Total lung resistance in response to acetylcholine challenge was measured in *Cav1*^*-/-*^ and WT mice at ages 1 month **(A)**, 3 months old **(B)**, 6 months old **(C)** and 12 months old **(D)**. The log of the dose of acetylcholine required for a 200% increase in airway resistance over the baseline (log PC200 in μg/mL) was calculated for each mouse **(E)**. Error bars indicate standard error. Significance was determined by 2 way ANOVA with a Bonferroni Post test **(A**-**D)** or a Student’s T test **(E)** using age matched *Cav1*^*-/-*^ and WT mice. n = 4-9 *p < 0.05. **P < 0.01 and ***P < 0.001 between WT and *Cav1*^*-/-*^*.*

Acetylcholine (Ach)-induced airway reactivity was assessed in *Cav1*^*-/-*^ and WT from ages 1 to 12 months. Over time AHR developed in *Cav1*^*-/*-^ mice. At one month old there was no difference between *Cav1*^*-/-*^ and WT mice in their airway response to Ach (Figure 1A, E). By 3 months of age *Cav1*^*-/-*^ mice show differences at high doses of Ach (Figure 1B), but no change in log PC200 airway reactivity (Figure 1E). By the time *Cav1*^*-/-*^ mice reached the age of 6 and 12 months AHR had clearly developed (Figure 1C, D, E). These findings suggest that *Cav1*^*-/-*^ mice developed AHR by 6 months of age independent of an external injury. We, therefore, investigated airway structural changes, which can contribute to AHR, taking place over time in the *Cav1*^*-/-*^ mice.

### Structural changes in the airways in aging ***Cav1***^-/-^ mice compared to WT animals

**Figure 2:**
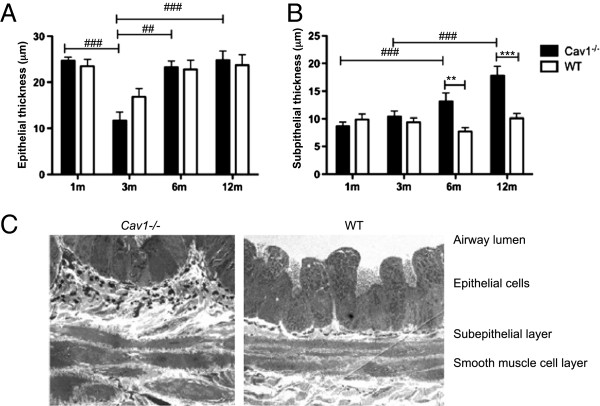
**Thickening of subepithelial basement membrane.** Using Trichrome-stained sections, morphometric analysis of thickness of bronchial epithelium **(A)** and thickness of subepithelial basement membrane **(B)** in *Cav1*^*-/-*^ and WT mice as they age. n = 8-17. Error bars indicate SEM. Significance between age was determined by one-way ANOVA with Tukey’s post test ##p < 0.01, and ###p < 0.001. Significance between age matched *Cav1*^*-/-*^ and WT mice was determined by Student’s T test. *p < 0.05, **p < 0.01. **(C)** TEM micrograph of the airway wall of a 3-month-old *Cav1*^*-/-*^ and WT mice. Magnification is 1,700x. Black staining represents elastic fibers.

**Figure 3:**
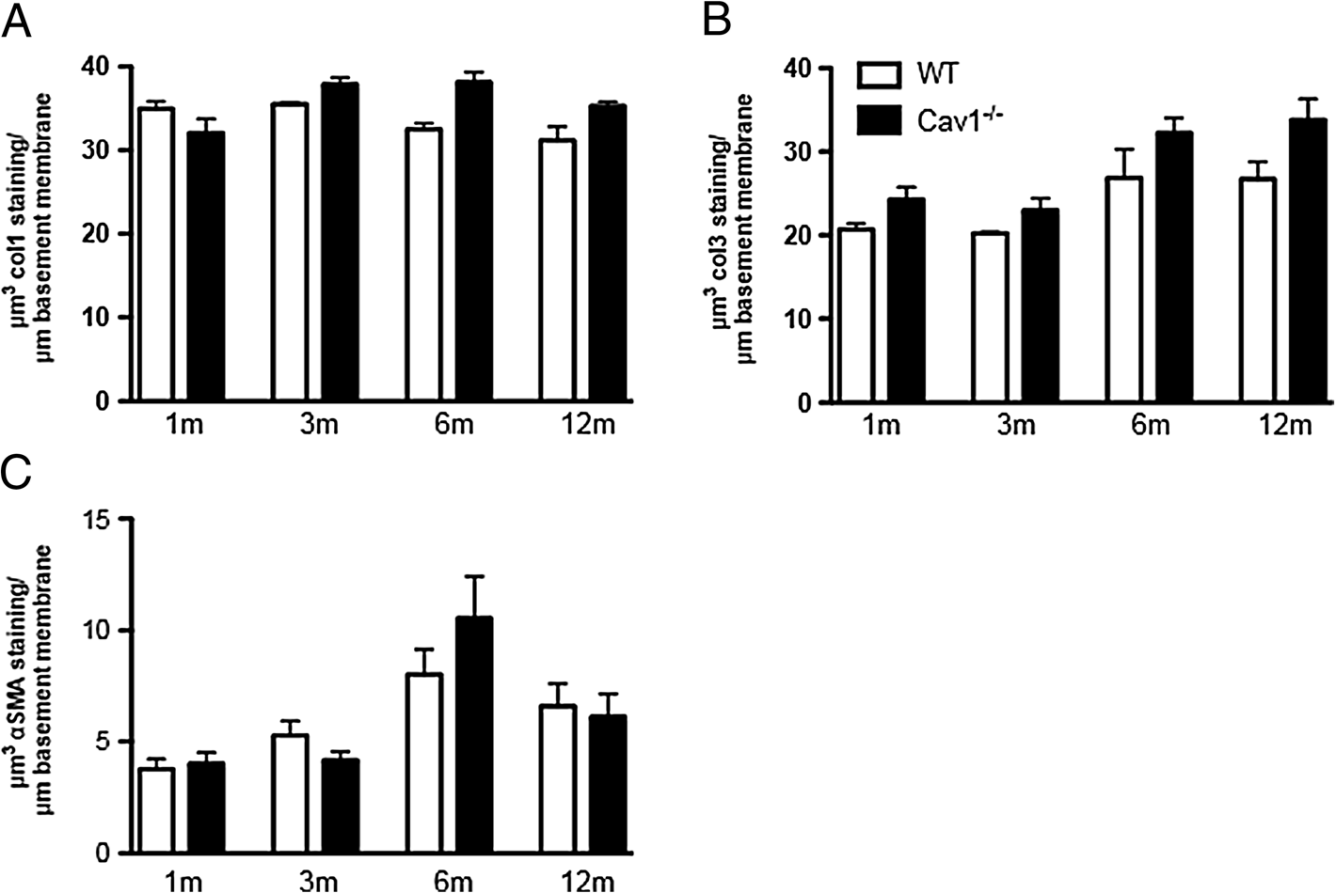
**Airway subepithelial collagen and smooth muscle layer thickness unchanged in *Cav1***^**-/- **^**mice.** Morphometric quantification of Collagen1 **(A)** and Collagen3 **(B)** immunostaining. Error bars indicate SEM. Significance was determined by ANOVA followed by a Tukey’s post test. **(C)** Morphometric analysis of thickness smooth muscle cell layer in *Cav1*^*-/-*^ and WT mice as they age. n = 14-21. Error bars indicate standard error. Significance determined by Student’s T test between age matched in *Cav1*^*-/-*^ and WT mice.

The following variables were examined as markers of structural changes in the airways: thickness of the epithelium and subepithelial basement membrane, subepithelial collagen deposition, and SMC thickening. To determine whether increased AHR was associated with early changes of airway remodeling, epithelial and subepithelial thickening were evaluated in all age groups. There was no significant difference in the epithelial thickness between *Cav1*^*-/-*^ and WT mice at any age (Figure 2A). In contrast, there was a significant increase in the thickness of the subepithelial matrix in 6- and 12-month-old *Cav1*^*-/-*^ mice compared to the WT mice as measured by morphometric assessment (Figure 2B) and supported by EM observations (Figure 2C). Whereas the subepithelial matrix thickness of WT mice did not significantly increase over time, *Cav1*^*-/-*^ mice showed a steady increase in the thickening of the subepithelial layer with significantly thicker subepithelial matrix at 12 months of age than at either 1 or 3 months. Immunostaining for collagen demonstrated no appreciable change in Type I or Type III Collagen (Figure 3A and B).

The α-SMA stained SMC layer increased in thickness as both *Cav1*^*-/-*^ and WT mice aged, peaking at 6 months of age, as measured by morphometric analysis (Figure 3C). However, the thickness of SMC layer surrounding the airway between *Cav1*^*-/-*^ and WT animals was never significantly different at any time point, indicating that the change in SMC development is a part of normal growth. This suggests that an increase in airway smooth muscle mass is not the cause of increased AHR in the aging *Cav1*^-/-^ mice.

This data indicate that cumulative thickening of the subepithelial matrix layer, but not an increase in smooth muscle mass, may contribute to the increase in AHR in *Cav1*^*-/-*^ mice over time.

### AHR after a 1-week OVA challenge in 2- and 6-month-old in ***Cav1***^-/-^ and WT mice

After it was established that 6-month-old *Cav1*^*-/-*^ mice had increased AHR and increased subepithelial matrix thickness, an OVA allergen challenge was carried out to examine the effect of previously established airway structural changes on AHR, airway remodeling and inflammation triggered by allergen challenge. Two-month old *Cav1*^*-/-*^ and WT mice received a 1-week OVA allergen challenge before airway remodeling is established. In addition, another group of mice, at 6 months of age, also received a 1-week OVA allergen challenged after we established the occurrence of airway remodeling in the knockout mice.

**Figure 4:**
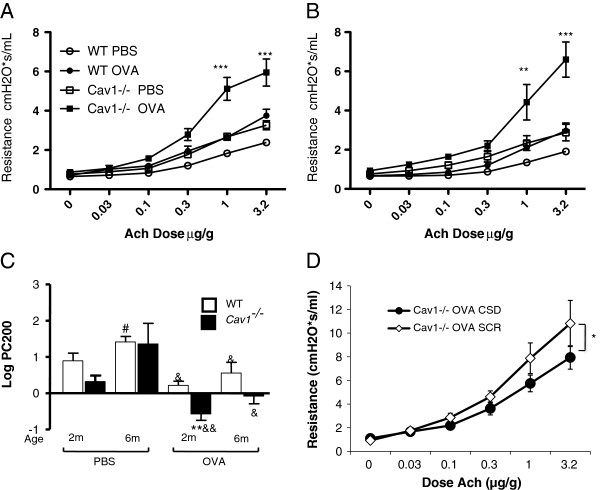
**Increased AHR in OVA-challenged *Cav1***^**-/- **^**mice and partial restoration with CSD.** Total lung resistance in response to acetylcholine challenge was measured in 2 month old **(A)** or 6 month old **(B)***Cav1*^*-/-*^ and WT mice after an OVA-allergen challenge. **P < 0.01, ***P < 0.001 between WT and *Cav1*^*-/-*^*. n = 4-10*. The Log PC200s were calculated from the dose-response curves of OVA challenged *Cav1*^*-/-*^ and WT mice **(C).** Significance between age was determined by one-way ANOVA with Tukey’s post test. Significance between age matched *Cav1*^*-/-*^ and WT mice was determined by Student’s T test. **P < 0.01 between WT and *Cav1*^*-/-*^ , #P < 0.05 between 2 and 6 months, &P < 0.05, &&P < 0.05 between PBS and OVA. Cav1 scaffolding domain (CSD), or a scrambled peptide (SCR) for control, was administered to *Cav1*^-/-^ mice in order to restore Cav1 function. An acetylcholine dose response curve was generated to analyze AHR **(D)**. *p < 0.05 by 2 way ANOVA, n = 8. All error bars represent standard error.

OVA-challenged 6-month-old mice had a lesser AHR response to allergen than their younger 2-month old counterparts independent of the presence of Cav1 (Figure 4C). However, both 2-month old and 6-month old *Cav1*^*-/-*^ mice developed more pronounced AHR than age-matched WT mice in response to allergen challenge. This indicates that the naturally-occurring airway structural changes in *Cav1*^*-/-*^ mice are not the only contributing factor to their more pronounced AHR after OVA challenge (Figure 4A-C).

Treatment of 2-month-old OVA-challenged *Cav1*^*-/-*^ mice with the Cav1 scaffolding domain (CSD) over a 4-week period prior to challenge was able to partially attenuate the exaggerated AHR in the *Cav1*^*-/-*^ mice, demonstrating the reversibility of these changes and the specific role of Cav1 (Figure 4D).

### Reduced inflammation after a 1-week OVA challenge in 6-month old ***Cav1***^-/-^ compared to WT mice

The degree of inflammation in 2- and 6-month-old OVA-challenged mice was compared to controls in both *Cav1*^*-/-*^ and WT groups to determine how these factors influenced AHR. There was no clear correlation between the inflammatory cytokine response and the severity of AHR. After OVA-challenge 2-month old *Cav1*^-/-^ mice had significantly higher levels of IL-4 in the BAL than WT mice (Table 1). In 6-month-old mice the inflammatory cytokine response to allergen challenge was less in the *Cav1*^*-/-*^ mice compared to WT with lower levels of IL-13 and IL-17 in BAL even though AHR in these mice remained higher than WT (Table 1).

**Table 1:** *BAL cytokine level comparisons between 2- and 6-month-old mice after a one-week PBS or OVA challenge*

		**WT**		***Cav1***^***-/-***^	
**Challenge**		**PBS**	**OVA**	**PBS**	**OVA**
2 M old	IL-4	10.5 ± 2	921 ± 446*	16 ± 7	1273 ± 1251###
	IL-5	94 ± 7	1711 ± 2596	128 ± 34	4434 ± 7265
	IL-9	13 ± 3	31 ± 12***	16 ± 3	25 ± 11
	IL-13	19.5 ± 9.5	122 ± 95	15 ± 5	129 ± 180**
	IL-17	36.5 ± 2	63 ± 24	38 ± 4	59 ± 24
	IgE	0	42 ± 74	0	49 ± 85
6 M old	IL-4	75 ± 13	341 ± 244	71 ± 10	520 ± 780
	IL-5	22 ± 3	903 ± 176	21 ± 5	542 ± 788
	IL-9	27 ± 3.5†	24 ± 14	24 ± 5	21 ± 5
	IL-13	34 ± 5.5	176 ± 121**	36 ± 5	45 ± 21##
	IL-17	84 ± 19	96 ± 16	82 ± 8	67 ± 14 ##
	IgE	0	1.8 ± 1.3	0	53 ± 68.9

*p < 0.05, **p < 0.01, ***p < 0.001 between PBS- and OVA-challenged mice. ##p < 0.01 ###p < 0.001 between *Cav1*^*-/-*^ and WT mice, †p < 0.05 between 2- and 6-month old mice. n = 6-12. Values are ng/ml ± standard deviation. Significance determined by one-way ANOVA with a Tukey post test.

In addition to measuring the BAL cytokine levels, the number and different types of inflammatory cells in the BAL were examined. There was no significant difference in the number of cells in the BAL of WT or *Cav1*^*-/-*^ OVA-challenged mice at 2- and 6 months of age (Table 2). The total BAL cell count in the 6-month-old OVA-challenged *Cav1*^*-/-*^ mice tended to be less than in the 2-month-old *Cav1*^*-/-*^ mice, however, this was also non-significant. These data indicate that in the *Cav1*^*-/-*^ mice, the increased AHR is not due to enhanced inflammatory response.

**Table 2:** *BAL inflammatory cell comparison between 2- and 6-month-old mice after a one-week PBS or OVA challenge*

	**2-month old**				**6-month old**			
	**WT**		***Cav1***^***-/-***^		**WT**		***Cav1***^***-/-***^	
	**PBS**	**OVA**	**PBS**	**OVA**	**PBS**	**OVA**	**PBS**	**OVA**
BAL Cells Cell/ml	0.8 ± 0.2	6.6 ± 1.5*	0.5 ± 0.2*	17.1 ± 5.3*	0.3 ± 0.1	3.4 ± 0.6*	0.3 ± 0.04	5.2 ± 2.5
Eosinophils%	6.1 ± 2	32.2 ± 2.4*	8.4 ± 1.6	38 ± 2.5*	1.3 ± 0.3	29.5 ± 7.1*	1.5 ± 0.7	19.4 ± 3.4*

Values are ± SEM *p < 0.05 between PBS- and OVA-challenged mice. Significance determined by one-way ANOVA with a Bonferoni post test.

### Enhanced collagen deposition and reduced mucus cells in aged OVA-challenged *Cav1*^*-/-*^ compared to WT mice

**Figure 5:**
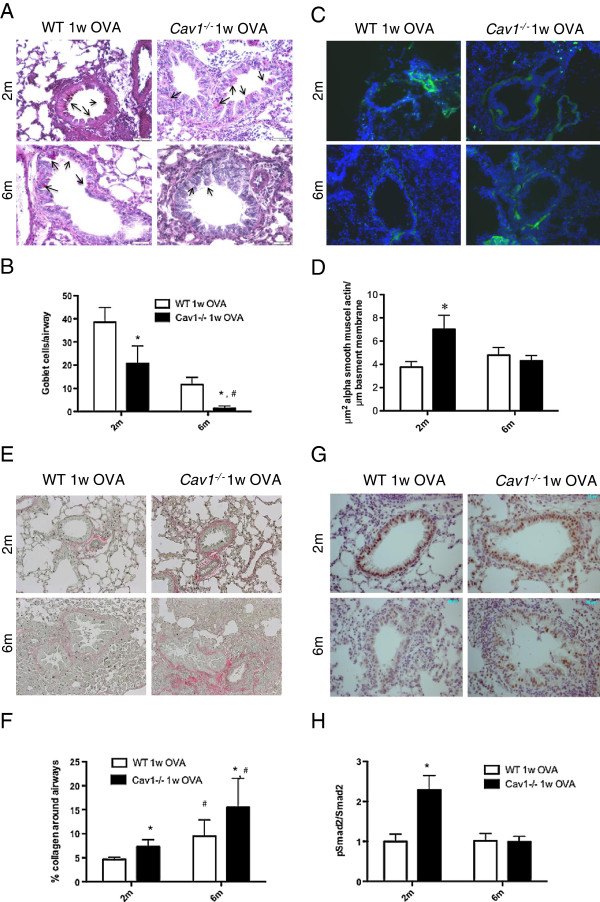
**Alterations of the airway remodeling features in mice after OVA challenge in *Cav1***^**-/- **^**mice.** Representative pictures of 1 week-OVA challenged WT and *Cav1*^*-/-*^ mouse lung sections. **(A**,**B**.**)** Goblet cells metaplasia was detected using Periodic Acid Schiff staining. Positive cells (indicated by arrows) were counted in airways of 100-200 μm in an average of 5 airways per animals is reported (Bar graph). **(C**,**D**.**)** Detection of α-SMA positive cells by immunohistochemistry and reporting the surface of positive staining relative to the length of basement membrane (Bar graph). **(E**,**F**.**)** Detection of collagen depositon around the airways using Picrosirius red staining and quantification of the relative amount of collagen deposition (Bar graph). **(G**,**H**.**)** Detection of pSmad2 using immunohistochemistry. Activation of the canonical TGF–β signaling pathway was also determined by western blot. Densitometric quantitations were carried out using the whole lung homogenates of WT and *Cav1*^*-/-*^ mice after a 1-week OVA challenge. Values are represented as relative protein levels of pSmad2 to total Smad2 (Bar graph). For all the measurement, * < p 0.05 represents significance by 2-way ANOVA between WT and *Cav1*^*-/-*^ mice for the same age group, and # < p 0.05 represents significance by 2-way ANOVA between same age group for WT and *Cav1*^*-/-*^ mice.

Features of airway remodeling following allergen challenge include goblet cell metaplasia, increased number of α–SMA positive cells, and collagen deposition. Following OVA allergen challenge, *Cav1*^*-/-*^ mice had less goblet cell metaplasia compared to WT mice at 2-month and 6-month (2-way ANOVA p-value = 0.0287, Figure 5A, B). Interestingly in both strains at 6-month, the response to OVA was associated with reduced goblet cell metaplasia (2-way ANOVA p-value = 0.0006), however, in absence of Cav1, the goblet cell population was barely detectable in 6-month-old OVA challenged mice. In contrast, we detected an increased thickness of α-SMA positive cells after OVA challenge in the 2-month old *Cav1*^*-/-*^ mice compared to WT. This increase in α-SMA and difference between *Cav1*^*-/-*^ and WT mice was not detected in the 6-month old animals (Figure 5C, D). Collagen deposition was also significantly increased in 2-month old *Cav1*^*-/-*^ mice compared to WT mice but was further enhanced in the 6-month old samples (Figure 5E, F).

As expected based on our previous studies, the level of activation of the canonical TGF-β signaling pathway was increased in 2-month *Cav1*^*-/-*^ mice after OVA challenge compared to WT mice as measured by immunohistology detection of pSmad2. Interestingly, the level of activation stayed similar in the OVA challenged WT mice compared to PBS controls but was dramatically reduced in the 6-month *Cav1*^*-/-*^ mice after OVA challenge (Figure 5G, H).

Overall, important differences in airway remodeling after OVA-challenge were measured between WT and *Cav1*^*-/-*^ mice and they appear to be diminished with age.

## Discussion

Murine models have been useful in studying the mechanisms that contribute to AHR [22-25]. The relative contribution of airway remodeling to AHR is difficult to assess as most of these models involve an allergic response triggering both inflammation and airway remodeling. In this study, we explored the inflammatory responses before and after airway remodeling was established. Our main finding was that *Cav1*^*-/-*^ mice develop AHR as they age. At 6 months of age, the AHR was significantly increased compared to WT mice, but not at an earlier age. Furthermore, there was also a significantly increased thickening of the subepithelial matrix layer in *Cav1*^*-/-*^ mice as compared to WT mice at 6 months of age. In previous studies, we have demonstrated by trichrome and picrosirius red staining that subepithelial collagen deposition is increased in *Cav1*^*-/-*^ mice by the time they are 6 months of age [17]. This suggests that AHR is related to collagen deposition in the bronchial subepithelial layer. Mathematical modeling has demonstrated that relatively minor increases in the thickness of the small airways can decrease the size of the airway caliber enough during muscle contraction to physiologically affect AHR, further supporting our findings [21]. We have also previously demonstrated that subepithelial collagen deposition contributes to increased AHR in a chronic OVA allergic airways disease model and that reversal of subepithelial and total lung collagen by relaxin treatment can reduce AHR [26].

The mechanisms for increased collagen deposition in the setting of Cav1 deficiency are incompletely understood. TGF-β promotes airway remodeling, especially promoting ECM deposition [10]. In asthmatics, TGF-β regulated by leukotrienes, can activate lung fibroblasts and increase collagen deposition [29,30]. We have previously established that Cav1 was involved in the regulation of TGF-β signaling in murine lungs and that over time, the absence of Cav1 leads to increased TGF-β signaling and collagen deposition in the parenchyma [17]. In this study we confirmed the increase in pSmad2 signaling in OVA challenged *Cav1*^*-/-*^ lungs. For the first time, we demonstrate the association of Cav1 deficiency with the development of increased AHR and thickening of the subepithelial layer. Cav1 interacts with and regulates a number of proteins in addition to TGF- β that are potentially important for regulation of AHR that we did not investigate in this study. For example, it has been well documented that Cav-1 participates in SMC contractility via Ca^2+^-dependent mechanisms. Indeed, SMC expressed Cav-1 and the caveolae contain a number of proteins that participate in the regulation of Ca^2+^[31-34]. Importantly, Sathish and al. [31] recently showed that Cav-1 regulates proteins (Orai1) that are important for calcium regulation. They also showed that an association between Cav1, calcium and inflammation.

After it was established that *Cav1*^*-/-*^ mice develop AHR and subepithelial thickness at 6 months of age, an OVA allergen sensitization and challenge was carried out at 2 months of age (before airway remodeling is established) and at 6 months of age (after airway remodeling is established) to explore differences in the inflammatory response. OVA challenge induced markedly elevated BAL cytokine levels in 2-month old WT and *Cav1*^*-/-*^ mice, but reduced cytokine levels in 6-month *Cav1*^*-/-*^ mice after the OVA allergen challenge. In a similar fashion, 6-month *Cav1*^*-/-*^ mice had a decreased number of inflammatory cells and percentage of eosinophils in the BAL after the OVA allergen challenge compared to 2-month *Cav1*^*-/-*^ mice. Increased number of mucous cells has been associated with increased IL-13, inflammation and eosinophilia [35]. Importantly, both age and Cav1 expression influence these parameters of response to allergen challenge. In our study, eosinophilia and levels of IL-5 decreased in older mice and correlated with reduced mucous cell metaplasia. Six-month-old OVA challenged *Cav1*^*-/-*^ mice reduced IL-13 in the BAL corresponding to few mucous cells. Few reports investigating airway inflammation in aged sensitized and challenged mice are available and even less on airway remodeling. The decrease in eosinophilia and overall cytokine level in aged mice both WT and *Cav1*^*-/-*^ mice was consistent with 2 of 3 previous studies using similar allergen challenge protocols [36-38]. Our findings suggest that the AHR observed in 6-month-old *Cav1*^*-/-*^ mice is independent of inflammation and more closely related to increased collagen around the airways. The work from Busse et al. also argues against a direct relationship between pulmonary inflammation or eosinophils and increased AHR, even though a different OVA sensitization and challenge protocol was used [36].

In asthmatic patients, the persistence of AHR can be associated with airway remodeling after the resolution of inflammation [6]. In addition, in mouse models, prolonged allergen challenge leads to persistent changes in the airways despite discontinuation of the allergen challenge [7,8]. These studies demonstrate a role of airway remodeling in AHR beyond the immediate inflammatory response. Other genetically engineered mouse models also point toward the importance of airway remodeling independent of inflammation [7,8]. For example, relaxin-deficient mice also show similar results, where an increase in airway remodeling leads to increased AHR [7,8,26].

The timing of airway remodeling development in asthma is an area of increasing interest. Evidence suggests that remodeling of airways may develop long before symptoms of asthma and inflammation appear [29]. In our study, older mice in both groups had less AHR and the BAL cytokines tended to be present at lower levels after the OVA allergen challenge. This suggests that some alterations in lung response to allergen challenge as the mice age were independent of the presence or absence of Cav1. Nevertheless, the reduction in inflammation was more prominent in the *Cav1*^*-/-*^ mice with greater reductions in eosinophils and IL-13. The decrease in inflammation in 6-month-old mice can account for the decrease in AHR seen in these mice compared to 2-month-old mice. Indeed, we demonstrated a decrease in the IL-4 levels in 6-month-old mice versus 2-month-old mice, and other studies have shown that treatment of sensitized mice with anti-IL-4 antibody prior to antigen stimulation reduces antigen-induced AHR, eosinophilia, and goblet cell metaplasia [39]. In addition, IL-4 can induce AHR and goblet cell metaplasia independent of IL-13 [31]. Despite the greater decrease in inflammation in the *Cav1*^*-/-*^ mice as they aged, the AHR of the *Cav1*^*-/-*^ remained greater than age-matched WT indication that remodeling rather than inflammation may play a role in the elevated AHR in 6-month-old OVA-challenged *Cav1*^*-/-*^ mice.

These findings have important implications, as they open avenues for possible therapeutic interventions to prevent the development of airway remodeling. Our data are supportive of the beneficial effects on airway remodeling after treatment with Cav1 scaffolding domain peptide. In addition, in other studies using relaxin-deficient mice, it has been demonstrated that treatment with relaxin is capable of reversing established airway remodeling and AHR [27]. These studies suggest that the development of anti-fibrotic therapies that could potentially prevent lung function decline associated with airway remodeling is warranted. This approach may be especially relevant in severe asthma, where well-established treatments with inhaled corticosteroids have limited efficacy in the control of airway remodeling development and reversibility [10,29].

Even though subepithelial fibrosis is an established characteristic in airway remodeling, other studies have demonstrated that changes in myocytes and globet cells may also induce AHR. In this murine model, we identified an increase in subepithilial thickening as the main contributor of AHR in *Cav1*^*-/-*^ mice, but because asthma is a heterogeneous disease, other cellular components may still play an important role in airway remodeling and AHR.

In conclusion, our findings demonstrate that Cav1 deficiency is associated with airway remodeling and the development of AHR. The airway remodeling progressed with age. Inflammation appears not to be required for the development of these age-related changes in Cav1 deficient mice. Cav1 may play a role, possibly through the TGF-β pathway, in the prevention of the development of subepithelial fibrosis and airway remodeling.

## Competing interests

They authors declare that they have no competing interests.

## Authors’ contributions

KEG: mouse work, lung functions, airway remodeling studies, interpretation of data, write up of the manuscript. SGR: morphometry analysis and write up of the manuscript. DJM: interpretation of data and write up of the manuscript. SKM: collagen analysis. MLKT: morphometry analysis and write up of the manuscript. CJLS: oversee of the entire project, interpretation of the results and write up of the manuscript. All authors read and approved the final manuscript.
